# Endobronchial Valve Treatment of Tuberculous Cavities in Patients with Multidrug-Resistant Pulmonary Tuberculosis: A Randomized Clinical Study

**DOI:** 10.3390/pathogens11080899

**Published:** 2022-08-10

**Authors:** Huiru An, Xiao Liu, Tianhao Wang, Lin Liu, Mengdie Yan, Jing Xu, Tao Wang, Wenping Gong, Zhongyuan Wang

**Affiliations:** 1 Tuberculosis Prevention and Control Key Laboratory/Beijing Key Laboratory of New Techniques of Tuberculosis Diagnosis and Treatment, Senior Department of Tuberculosis, The Eighth Medical Center of PLA General Hospital, Beijing 100091, China; 2 Department of Emergency, The Eighth Medical Center of PLA General Hospital, Beijing 100091, China; 3 National Clinical Research Center for Infectious Disease (Shenzhen), Guangdong Provincial Clinical Research Center for Infectious Diseases (Tuberculosis), Shenzhen Third People’s Hospital, Southern University of Science and Technology, Shenzhen 518112, China

**Keywords:** pulmonary tuberculosis (PTB), multidrug-resistant pulmonary tuberculosis (MDR-PTB), endobronchial valves (EBVs), cavity closure, efficacy

## Abstract

Background: Multidrug-resistant pulmonary tuberculosis (MDR-PTB) has become a major cause of high morbidity and mortality related to TB. Conventional drug regimens are ineffective for the treatment of MDR-PTB patients with cavities. This study aimed to evaluate the clinical efficacy and safety of one-way endobronchial valves (EBVs) for the treatment of cavities in MDR-PTB patients. Methods: MDR-PTB patients with positive sputum cultures, sputum smears, and cavities were treated with EBVs in the drainage bronchus of the pulmonary cavity between November 2013 and March 2018. The participants comprised those who had failed previous anti-tuberculosis therapy, as determined by drug susceptibility testing. Results: Thirty-five MDR-PTB patients were included, three of whom were lost during follow-up. The size of the lung cavity was reduced in all of the patients after EBV implantation, including the three lost to follow-up. In the remaining 32 patients, the sputum culture conversion (SCC) rate reached 100%, and the cavity closure rate was 68.8%. There were no significant differences in the cavity closure rate between patients aged ≤40 and >40 years, between the upper and lower lobes, or between the use and non-use of linezolid groups (*p* > 0.05). Interestingly, the cavity closure rate was higher in women than in men (*p* = 0.005). Moreover, the cavity closure rate correlated with the time to SCC (correlation coefficient, 0.8933; *p* < 0.0001). There were no severe adverse events in the patients treated with EBV implantation. Conclusion: EBV installation is effective and safe for the treatment of cavities in MDR-PTB patients. The efficacy of EBV treatment may not be affected by age, disease course, or the location of the lung lobe in the cavity.

## 1. Introduction

Drug-resistant tuberculosis (DR-TB) is a serious global public health concern. According to the Global Tuberculosis Report released by the World Health Organization (WHO) in 2021, the number of new cases of TB worldwide in 2020 was as high as 9.87 million, and nearly 500,000 cases developed Rifampin-resistant tuberculosis (RR-TB), of which 78% were multidrug-resistant pulmonary tuberculosis (MDR-PTB) [1]. Patients with DR-TB or MDR-PTB often have single or multiple lung cavities. These cavities have thick walls and insufficient blood circulation. Therefore, it is difficult for anti-tuberculosis drugs to enter them, making the *Mycobacterium tuberculosis* (*M. tuberculosis*) hidden in the cavities unable to be killed by these drugs and host immunity, and thus prolonging TB or even making it incurable [2,3]. Moreover, a previous study found that *M. tuberculosis* strains isolated from lung cavities were more resistant than those isolated from sputum samples, suggesting that cavities play a vital role in the evolution of the drug resistance of tuberculosis [2]. Therefore, treating and closing the pulmonary TB cavity has become key to controlling DR-TB. Various methods that rely on external forces to compress lung tissue have been used to seal cavities, including artificial pneumothorax and pneumoperitoneum [4,5]. However, these methods entail many adverse events that are difficult for patients to tolerate, seriously affecting their quality of life, and have been discontinued.

**Figure 1:**
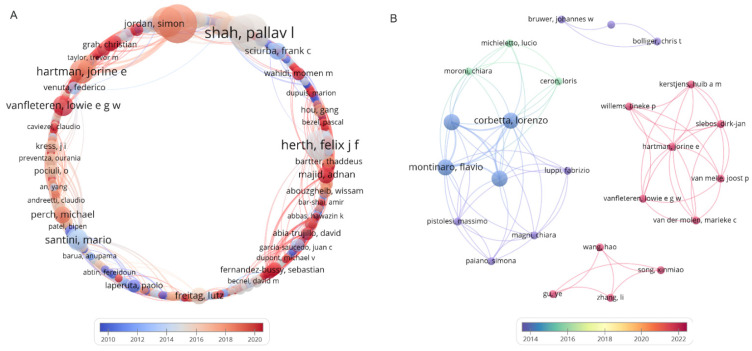
Studies on EBV treatment in the PubMed database. (**A**) Co-authorship map of endobronchial valve (EBV) treatment. (**B**) Co-authorship map of EBVs for the treatment of TB. Both co-authorship maps based on bibliographic data obtained from PubMed (https://pubmed.ncbi.nlm.nih.gov/) are visualized by using the VOSviewer version 1.6.16, which was developed by Nees Jan van Eck and Ludo Waltman at Leiden University’s Centre for Science and Technology Studies CWTS. The search terms used in PubMed were “EBVs” or “endobronchial valves” and/or “tuberculosis” in the title or abstract. We included studies from 1990 to 2022, and used default values for the other parameters. The data were collected on 8 July 2022.

In recent years, one-way endobronchial valves (EBVs) have been used to treat persistent air leaks, emphysema, pneumothorax, bronchocutaneous fistula, and bronchopleural fistula owing to their advantages of causing only minor trauma and having fewer complications; they have achieved sound therapeutic effects [6,7,8,9,10,11,12,13,14,15] (Figure 1A). The principle underlying EBVs is to seal the drainage bronchus through a one-way valve so that the air can exit but not enter, promoting the volume reduction of the corresponding lung tissue without affecting the secretion drainage [16]. As an emerging technology, five reports on EBV were found in the PubMed database (Figure 1B), of which only two studies worldwide reported the safety and efficacy of EBV in the treatment of pulmonary cavities in MDR-PTB patients. As early as 2016, Corbetta et al. treated cavitary TB using EBV to induce lobar volume reduction, and found that the cavities completely collapsed in four of five patients after EBV treatment without short-term severe or long-term complications, suggesting that EBV may be a potential treatment for cavities in MDR-PTB patients [17]. Coincidentally, in the same year, researchers from Russia also conducted a study on EBV for the treatment of pulmonary cavities in 49 MDR-PTB patients who had failed previous anti-tuberculosis drug treatment, and found that EBV treatment could shorten the time to sputum culture conversion (SCC) and enhance the cavity closure rate [18].

To our knowledge, this study is the first in China to evaluate the safety and efficacy of EBV in the treatment of pulmonary cavities in MDR-PTB patients. This study is also the third international study after studies from Italy and Russia to evaluate EBV for the treatment of pulmonary cavities in MDR-PTB patients. Herein, we assessed the efficacy and safety of EBV in the treatment of cavities in MDR-PTB patients. Our results indicate that EBV treatment could promote the closure of cavities and shorten the time to SCC. Moreover, we demonstrate new evidence for the evaluation of the efficacy and safety of EBV in treating cavities in MDR-PTB patients, and we provide novel ideas for controlling MDR-PTB.

## 2. Results

### 2.1. Baseline Characteristics

Thirty-five MDR-PTB patients with poor anti-tuberculosis treatment status were recruited, including 22 men and 13 women. The median age was 30 (range, 26–38) years, and the median disease duration was 32 (range, 22–60) months. For statistical convenience, the number of treated cavities was calculated according to the lobe in which the EBV was implanted for multiple cavities in a single lobe. Fifty-four cavities were treated in 35 patients, and 94 EBVs were implanted. Briefly, three cavities were treated with EBVs in four patients, two cavities were treated with EBVs in 12 patients, and one cavity was treated with EBV in 19 patients. After EBV treatment, three female patients with six cavities and 10 EBVs were lost to follow-up. Ultimately, 32 patients completed EBV treatment and discontinued anti-tuberculosis drugs. Forty-eight cavities were treated, including 33 in the upper lobe and 15 in the lower lobe. Follow-up for three years after drug withdrawal showed that five patients relapsed, including patients demonstrating a recurrence of the original cavities and three patients presenting new cavities and lesions outside initial lung lesions.

### 2.2. Correlations among 11 Variates in MDR-PTB Patients Treated with EBVs

**Figure 2:**
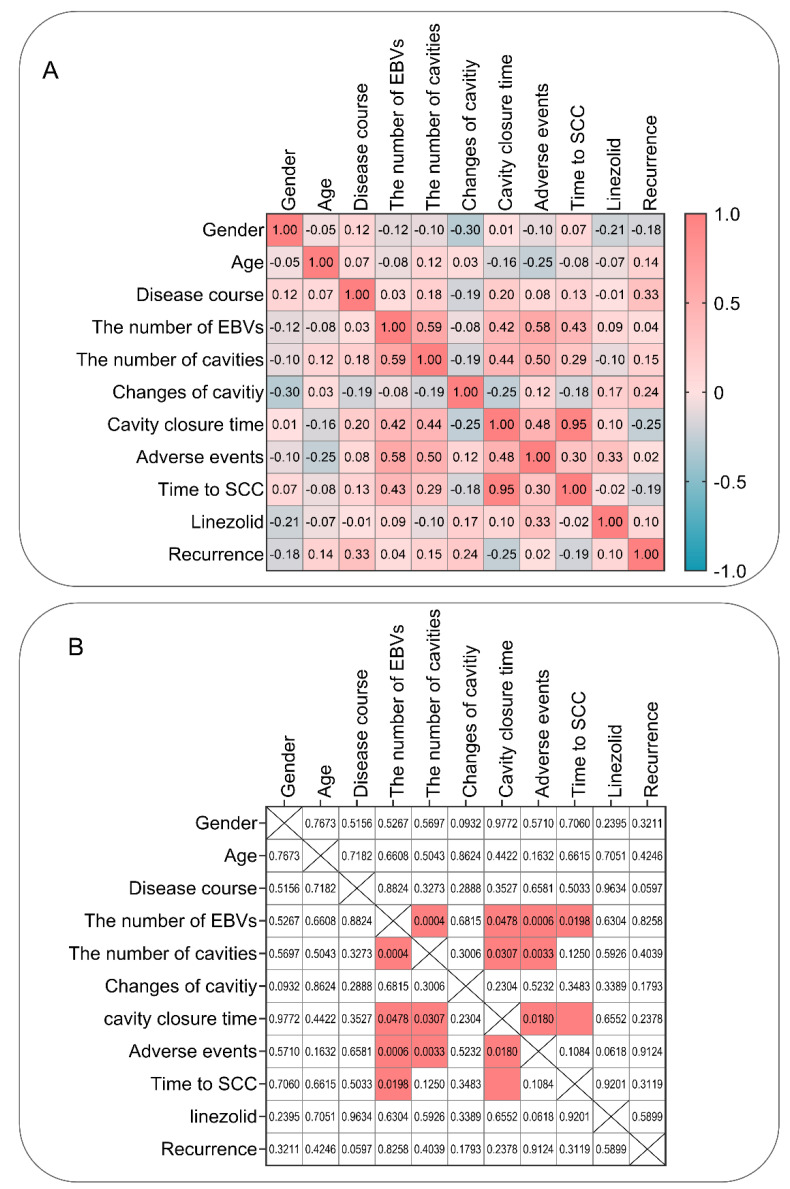
Heatmap of the correlation matrix among 11 variates in MDR-PTB patients treated with EBVs. (**A**) The heatmap of the Pearson r values. The r values from −1 to 1 are shown in blue to red. (**B**) The heatmap of the *p* values (*p* < 0.05, red).

Correlations among sex, age, disease course, number of EBVs, number of cavities, changes in cavity, cavity closure time, adverse events, time to SCC, linezolid use, and recurrence (Table S1) were determined using Pearson’s correlation coefficient. Our study found that (Figure 2) (1) the number of EBVs significantly correlated with the number of cavities (Pearson r = 0.589; *p* = 0.0004), cavity closure time (Pearson r = 0.417; *p* = 0.0478), adverse events (Pearson r = 0.576; *p* = 0.0006), and time to SCC (Pearson r = 0.430; *p* = 0.0198); (2) the number of cavities was significantly correlated with cavity closure time (Pearson r = 0.442; *p* = 0.0307) and adverse events (Pearson r = 0.496; *p* = 0.0033); and (3) cavity closure time was significantly correlated with adverse events (Pearson r = 0.479; *p* = 0.0180) and time to SCC (Pearson r = 0.953; *p* = 2.0976 × 10^−12^).

### 2.3. Time to Cavity Closure and SCC in MDR-PTB Patients after EBV Treatment

**Figure 3:**
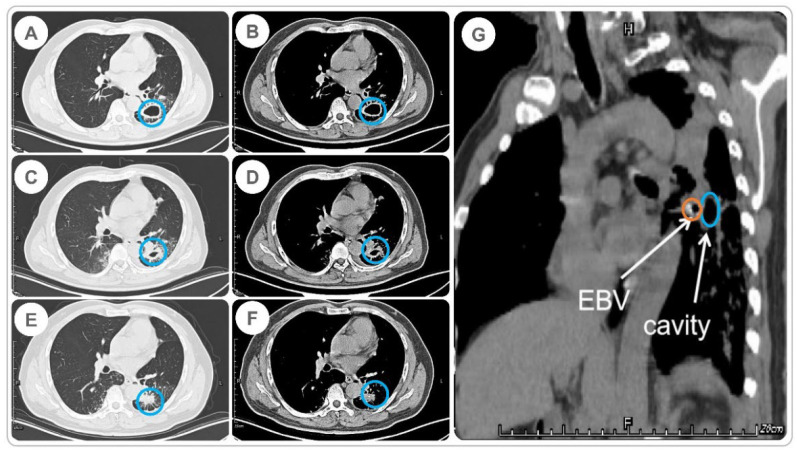
Cavity changes before and after EBV implantation in an MDR-PTB patient. (**A**) Cavity size (lung window) before EBV implantation on 2 August 2017; (**B**) size of the cavity (mediastinal window) before EBV placement on August 2, 2017; (**C**) size of the cavity (lung window) four days after EBV implantation on 14 August 2017; (**D**) size of the cavity (mediastinal window) four days after EBV implantation on 14 August 2017; (**E**) size of the cavity (lung window) 60 days after EBV implantation on 18 October 2017; (**F**) size of the cavity (mediastinal window) 60 days after EBV implantation on 18 October 2017; and (**G**) the EBV and cavity in the mediastinal window. The cavity and implanted EBV are highlighted with blue and yellow circles, respectively.

The size of the cavities in the 35 MDR-PTB patients decreased after treatment compared to their pre-treatment sizes. After excluding three patients lost to follow-up, 48 cavities were treated in the remaining 32 patients, 33 (68.8%) of which were closed, seven (14.6%) of which were markedly effective, and eight (16.7%) of which were effective, with an overall effectiveness of 100%. Furthermore, we determined the time of cavity closure in 32 MDR-PTB patients. In the case of multiple cavities in a single lobe, the cavity closure time was calculated based on the last cavity closure time. The median time to cavity closure was 11 (range, 2–15) months. Surprisingly the SCC rate of patients treated with EBV was as high as 100%, and the median duration of SCC was 16 (range, 5–23) months. Figure 3 shows the changes in the pulmonary cavities in an MDR-PTB patient before and after EBV treatment.

### 2.4. Comparisons of the Cavity Closure Rate in Different Groups Divided by Clinical Parameters in 32 MDR-PTB Patients

Univariate analysis was performed in order to investigate the effects of age, sex, linezolid, and pulmonary lobe on cavity closure after EBV treatment (Table 1). Our results showed the following: (1) Age: Thirty-two patients with complete follow-up records were divided into ≤ 40-year-old and > 40-year-old groups. There were 26 MDR-PTB patients in the ≤ 40-year-old group, in whom 27 (71.1%) cavities were closed after EBV treatment and one (28.9%) was not closed; in the > 40-year-old group, there were six MDR-PTB patients, in whom six cavities (60.0%) were closed and four (40.0%) were not. Statistical analysis revealed no significant difference in cavity closure between both groups after EBV treatment (*p* = 0.7028; odds ratio [OR] [95% confidence interval [CI] = 1.6360 [0.4465–6.1990]; relative risk [RR] [95% CI] = 1.1840 [0.7718–2.3270]). (2) Sex: Twenty-two male patients completed EBV treatment; 17 of 31 (54.8%) cavities were closed and 14 (45.2%) were not. Ten female patients completed EBV treatment; 16 of 17 (94.1%) cavities were closed and one (5.9%) was not. Comparing the number of closed cavities between male and female patients, the cavities in male patients were less likely to close than those in female patients after EBV treatment (*p* = 0.0078; OR [95% CI] = 0.0759 [0.0068–0.5555]; and RR [95% CI] = 0.5827 [0.3969–0.8204]). (3) Linezolid: Based on the use of linezolid, 32 MDR-PTB patients were divided into linezolid-free and linezolid groups. There were 17 MDR-PTB patients in the linezolid-free group; 15 of 26 (57.7%) cavities were closed, and 11 (42.3%) were not. There were 15 MDR-PTB patients in the linezolid group; 18 of 22 cavities (81.8%) were closed, and four (18.2%) were not. Statistical analysis showed that the cavity closure rate was lower in the group without linezolid, but the difference was not statistically significant (*p* = 0.1179; OR [95% CI] = 0.3030 [0.0933–1.0490]; RR [95% CI] = 0.7051 [0.4614–1.0350]). (4) Lung lobe: According to the location of the cavity, 27 and 14 MDR-PTB patients were divided into upper and lower lobe groups; patients with both upper and lower lobe cavities were counted separately. A total of 27 MDR-PTB patients were included in the upper lobe group; 22 (66.7%) of 33 cavities were closed, and 11 (33.3%) were not. Moreover, 14 MDR-PTB patients were included in the lower lobe group; 15 (73.3%) cavities were closed, and four (26.7%) were not. Statistical analysis revealed no significant difference in the number of cavity closures between both groups (*p* = 0.7458; OR [95%CI] = 0.7273 [0.2165–2.6800]; RR [95% CI] = 0.9091 [0.6274–1.4510]).

### 2.5. Correlation between Cavity Closure Time and Disease Course or Time to SCC

**Figure 4:**
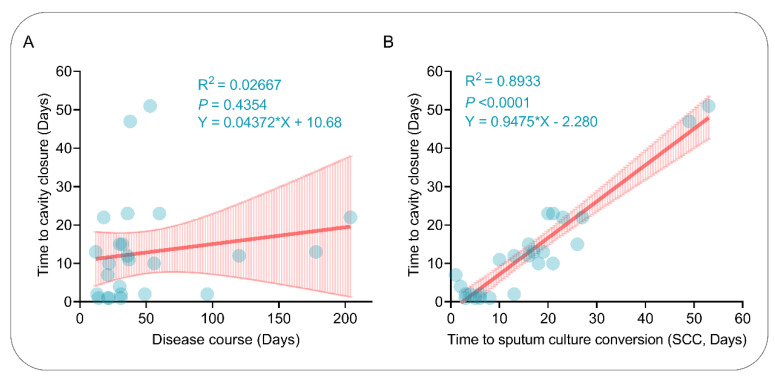
Simple linear analysis between the time to cavity closure and the disease duration (**A**) or time to SCC (**B**). Data for each patient were represented by blue dots, fitted linear relation-ships were represented by thick red lines, and error bars on both sides were represented by thin red lines.

Simple linear regression was performed in order to explore the potential correlation between the cavity closure time and the disease course or the time to SCC. Our results indicated no correlation between the cavity closure time and disease course (Figure 4A; R^2^ = 0.02667; *p* = 0.4357). Interestingly, the time to cavity closure positively correlated with the time to SCC (Figure 4B; R^2^ = 0.8933; *p* < 0.0001); for multiple cavities in a patient, the time to cavity closure was calculated according to the time to the last closed cavity. These results suggest that the earlier the sputum mycobacteria turn negative, the more beneficial the therapeutic effect in MDR-PTB patients.

### 2.6. Adverse Events in EBV Therapy

In order to evaluate the safety of EBV treatment in MDR-PTB patients, we analyzed the incidence of adverse events associated with EBV treatment. A total of 13 adverse events occurred (Table 2), including (1) fever in three patients (accounting for 23.1% of adverse events), which recovered after anti-infective treatment; (2) granulomas around the EBV in six patients (46.2%), which were eliminated by cryotherapy and did not recur; (3) one patient developed chest tightness due to pulmonary disease, and the symptoms improved after EBV removal; (4) one patient had a small amount of hemoptysis, but no further hemoptysis occurred after hemostatic treatment; (5) the EBV was displaced and embedded in one patient, and could not be removed, but no other adverse events occurred during the three-year follow-up; and (6) in one patient, EBV was detached due to insufficient depth, and did not fall off again after EBV replacement.

## 3. Discussion

The treatment of MDR-PTB has always been a challenge in TB control. The current therapeutic schedule is dominated by a complex anti-tuberculosis treatment regimen comprising multiple drugs. This treatment regimen is expensive and has many adverse effects, inducing poor patient compliance and significantly affecting its efficacy. Since 2016, the WHO guidelines have recommended a short-term regimen of 9–12 months instead of an individualized regimen of at least 20 months for the treatment of MDR-PTB [19]. However, treating MDR-PTB remains difficult, and is closely related to the pulmonary cavity caused by *M. tuberculosis* infection [20].

Pulmonary cavity lesions often accompany MDR-PTB patients, and there is a significant amount of caseous necrosis and *M. tuberculosis* on the inner wall of the cavity, which is the primary source of *M. tuberculosis* dissemination in vivo. Some cavities have thick walls and insufficient blood circulation, making it difficult for anti-tuberculosis drugs to penetrate the cavity and kill *M. tuberculosis*. Thus, these cavities will become natural “refuges” for *M. tuberculosis*, making this type of TB, as well as persistent TB, develop drug resistance. Although *M. tuberculosis* can grow freely in the cavity, we found its weakness. *M. tuberculosis* is a facultative aerobe. Therefore, promoting cavity closure can create a local hypoxic environment that is not conducive to *M. tuberculosis* reproduction, providing a potential method for curing MDR-PTB. Zephyr (PulmonX Inc., Redwood, CA, USA) EBV has a duckbill-like configuration comprising a one-way flap valve, a self-expanding nickel-titanium memory alloy stent, and silicone covering the inside and outside of the stent [21]. It is predominantly designed for lung volume reduction in emphysema, and is not readily associated with obstructive inflammation [21]. As early as 2015, Klooster et al. treated 34 patients with emphysema without interlobar collateral ventilation using EBVs. They found that, compared with the control group, pulmonary function—including forced expiratory volume in the first second (FEV1)—was significantly improved in the EBV group [22]. Similarly, a randomized controlled trial evaluated the efficacy and safety of EBVs in 93 patients with homogeneous emphysema in the absence of collateral ventilation, and the results have shown that the improvements in FEV1 from baseline were 13.7% ± 28.2% and −3.2% ± 13.0% in the EBV and control groups, respectively [23]. Previous studies have demonstrated that EBV can improve lung function, exercise tolerance, and quality of life in patients with homogeneous emphysema without collateral ventilation.

In this study, unidirectional EBV was implanted in the cavity-draining bronchus to treat the cavities of 35 MDR-PTB patients who had previously failed anti-TB treatment. Excluding three patients who were lost to follow-up, the remaining 32 patients were treated with EBVs. Our results have indicated that the size of all of the 48 cavities was reduced compared to that before EBV treatment, the cavity closure rate reached 68.8%, and the negative conversion rate of sputum mycobacteria reached 100%. The study of Corbetta et al. from Italy used a flexible bronchoscope for unidirectional implant EBV to treat four cavities in three MDR-PTB patients and one cavity in one patient with refractory PTB, as a result, four (80%) cavities closed after treatment [17]. The aforementioned study has many similarities with ours: both were operated using a flexible bronchoscope, and the treatment of cavities through EBV in MDR-TB patients achieved good results, suggesting that EBV implantation can be used for the treatment of cavities in MDR-PTB patients. Moreover, Levin et al. applied one-way EBV (MedLung, certified in Russia and Europe) to treat cavities in 49 MDR-PTB patients who had failed previous treatment; they found that the sputum negative conversion rate was 95.9%. The cavity closure rate was 95.9% [18]. However, among the 53 cases in the control group receiving second-line anti-tuberculosis drugs only, the sputum negative conversion rate and cavity closure rate were only 37.7% and 20.7%, respectively. Moreover, the cavity closure time was significantly longer than that in the EBV group [18]. All of the of these studies suggest that the one-way bronchial valve implanted in the cavitary drainage bronchus can indeed promote the closure of cavities in MDR-PTB patients, enhance the sputum negative conversion rate, and improve the cure rate of MDR-PTB. Unlike an EBV, which can be placed under local anesthesia using a flexible bronchoscope, an EbV is a hollow cylinder made of inert medical rubber composite material that needs to be placed under general anesthesia and a rigid bronchoscope. Therefore, an EBV can be more conveniently implanted. However, owing to limited data, it is difficult to distinguish the efficacy between EBV and EbV implantations in treating cavities in MDR-PTB patients.

Our study found that the time of cavity closure was positively correlated with the time to SCC. The earlier the cavity was closed, the earlier the sputum turned negative, suggesting that for patients with cavitary MDR-PTB, EBV implantation should be performed as soon as possible before anti-tuberculosis drug therapy failures, which may significantly promote the cavity closure and considerably shorten the treatment course of MDR-PTB. Moreover, our study also found that in 32 patients who completed a course of EBV treatment, the cavity closure rate was not statistically different between the age groups and the upper and lower lobes. The cavity closure time was not correlated with the disease course. These results indicate that the selection of indications for minimally invasive interventional therapy might not be limited by the patient age, distribution of lobes, and disease course. In this study, female patients had a high cavity closure rate, possibly due to the small sample size of the study or smoking, diet, genetics, and other factors. Furthermore, in this study, it was found that the cavity closure rate of patients taking linezolid was slightly higher, but there was no statistical difference compared to non-users, which may be related to the impact of the small sample size on the statistical results. Therefore, it is necessary to increase the sample size in order to further clarify the impact of the application of new anti-tuberculosis drugs, such as linezolid, on cavity closure.

Moreover, we evaluated the recurrence in 32 MDR-PTB patients treated with EBV. We found that five (15.6%) patients experienced recurrence. Four patients had increased lesions or enlarged cavities at the original site. This recurrence may be related to the resurgence of endogenous *M.* tuberculosis caused by the short duration of the anti-tuberculosis drug treatment after cavity closure. Moreover, the recurrence rate of MDR-PTB in patients after EBV treatment in this study was higher than that reported by Levin et al. [18], which may be related to the fact that a considerable number of MDR-PTB patients in their study chose to surgically remove the diseased site after EBV implantation.

In order to assess the safety of EBV treatment, we counted the adverse events that occurred in patients after EBV treatment. In this study, only a few patients had complications—such as fever, granuloma around the valve, chest tightness, hemoptysis, and valve displacement and insertion—that could not be removed, along with the valve falling off. Moreover, none of the patients experienced serious adverse events, including severe infection or pneumothorax. In a previous study, the safety of the Zephyr^®^ EBV was confirmed in four MDR-PTB patients and one patient with atypical mycobacteria. There were no severe short- or long-term complications [17]. Similarly, Klooster et al. also reported the safety of EBV in the treatment of cavities in 34 patients with MDR-PTB; 23 and five serious adverse events were observed in the EBV and control groups during the six-month follow-up, respectively [22]. It should be noted that most of the participants in Klooster’s study had emphysema and pneumothorax. The anatomical and pathophysiological characteristics of the patient’s lungs may increase the probability of the abovementioned adverse events; therefore, the natural rate of adverse events caused by EBV treatment in this study may have increased owing to the patient’s complications.

The greatest value of this study is that it presents the association of multiple parameters with EBV treatment, which will provide future case-control studies with endpoints that can be relied upon for multivariate analysis. This study provides new insights into the more precise definition of the predictors of improved MDR-PTB treatment outcomes. Additionally, this study had some limitations: (1) the sample size was small, which might have affected the accuracy of the EBV treatment results; (2) no control group was included, and all of the 34 MDR-PTB patients were treated with EBV; (3) EBV instillation is an expensive, time-consuming, and resource-intensive treatment option, and its widespread implementation in countries with high TB burdens faces many challenges, such as low incomes, poor healthcare resources, a lack of specialized equipment, and a lack of physicians proficient with the technology.

## 4. Materials and Methods

### 4.1. Study Design and Ethics Statement

This self-controlled prospective clinical study was conducted at the Eighth Medical Center of PLA General Hospital (Beijing, China). This clinical study was approved by the Ethics Committee of the Eighth Medical Center of PLA General Hospital (approval number: 2015ST005), and was conducted in accordance with the ethical standards of the Declaration of Helsinki. This clinical study was registered in the Chinese Clinical Trial Registry (registration number: ChiCTR2200059081).

### 4.2. Participants and Inclusion and Exclusion Criteria

MDR-PTB patients with cavities hospitalized in the Senior Department of Tuberculosis of the Eighth Medical Center of the PLA General Hospital between November 2013 and March 2018 were enrolled according to the order of hospitalization. The MDR-PTB patients with cavities were diagnosed under the guidelines of the “Diagnosis for pulmonary tuberculosis (WS 288-2017)” [24] and “Classification of Tuberculosis (WS 196-2017)” [25], which were approved by the National Health and Family Planning Commission of China.

Moreover, the inclusion criteria of MDR-PTB patients with cavities were as follows: (1) an age between 18 and 70 years; (2) no severe cardiopulmonary dysfunction; (3) a positive acid-fast bacillus (AFB) smear or sputum smear; (4) based on the results of the phenotypic drug sensitive test (DST) and previous medication history, individualized anti-tuberculosis treatment for six months deemed ineffective as the patient had no changes in the size of the cavity and SCC; (5) the patients could voluntarily participate in this clinical study and sign informed consent; and (6) there were no contraindications to bronchoscopy.

The exclusion criteria were as follows: (1) an age over 75 years or under 18 years; (2) cardiac insufficiency or a history of severe heart diseases such as coronary heart disease, rheumatic heart disease, pulmonary heart disease, and so on; (3) severe active pneumonia, hemoptysis, or severe pulmonary diseases such as emphysema and bullae that affect valve placement and respiratory function; (4) congenital or acquired malformation and deformation of the bronchus that affect valve placement; (5) caseous pneumonia with dissolved cavities and decay cavities; (6) severe liver and kidney damage affecting anti-tuberculosis treatment; and (7) other situations where valve implantation is not suitable.

### 4.3. Clinical Treatment Regimen

**Figure 5:**
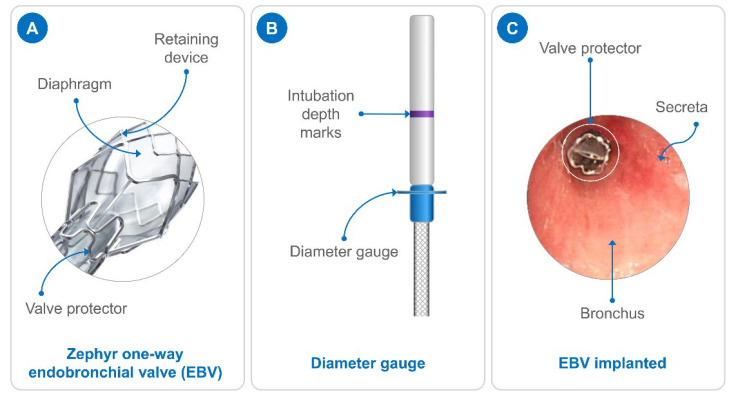
Schematic diagram of EBV treatment. (**A**) Structure diagram of a Zephyr one-way EBV; (**B**) diameter gauge; and (**C**) EBV was implanted using a bronchoscope.

All of the enrolled patients continued to receive systemic individualized anti-tuberculosis treatment according to their phenotypic DST results and previous medication history. The selected anti-tuberculosis drugs included isoniazid, rifampicin, pyrazinamide, ethambutol, rifapentine, levofloxacin, moxifloxacin, linezolid, protionamide, and clarithromycin. The EBV was placed at the corresponding segment or subsegmental opening of the TB cavity, draining the bronchus (Figure 5A). The patients were followed-up in order to determine whether the EBV was displaced, and to evaluate local airway and lung cavity changes and the results of the AFB smear and conversion of sputum smear from positive to negative.

### 4.4. EBV Placement Method

Potential cavities in the lungs were determined using high-resolution computed tomography. The patient then inhaled aerosolized lidocaine through the airway. A flexible bronchoscope (Olympus BF-1TQ180, Tokyo, Japan) with a 2.8-mm working channel was used to observe the trachea, bilateral main bronchi, and lobar bronchi, and to clear the airway secretions. Next, the bronchoscope was placed in the target lung lobe or segment in order to observe the shape and size of the lumen, and to estimate the number of EBVs (Zephyr, PulmonX Inc., Redwood, CA, USA) that needed to be placed. A diameter gauge (Figure 5B) was used to measure the diameter of the target segment or subsegment bronchia, and to determine the size of the desired EBV. There are two sizes of EBVs: 4 mm, with an available size range of 4.0–7.0 mm, and 5.5 mm, with a suitable size range of 5.5–8.5 mm.

In order to evaluate the target bronchial diameter and estimate the number of EBVs required, the EBV in the loader was transferred into the valve delivery device through the valve inserter. The bronchoscope was withdrawn to the lower trachea and an EBV-equipped delivery device was inserted through the working hole. The delivery device was sent to the opening of the bronchus, and the marker line of the delivery device was flushed with the edge of the ridge. Finally, a push rod was pushed to release the EBV into the center of the bronchial opening (Figure 5C). The position and function of the EBV were confirmed using a bronchoscope. If an EBV’s position and function were appropriate, other EBVs would be sequentially placed according to the abovementioned steps. Otherwise, the steps were repeated until the EBV placement was successful. Finally, the position and function of each EBV was confirmed once again, and the bronchoscope was gently exited after clearing the secretions in the airway. One day later, the position of the EBVs was determined again using chest computed tomography (CT).

### 4.5. Follow-Up Observation and Efficacy Judgment

Bronchoscopy and chest CT were reviewed in the first, second, and third months after EBV placement and every 2 or 3 months after the third month. AFB smears and the conversion of sputum smears from positive to negative were also determined at each review. Furthermore, the location and function of each EBV, as well as potential granulomas around the EBV, were assessed. After observation and confirmation, the sputum attached to the EBV surface was cleaned using a bronchoscope. Notably, changes in pulmonary cavities and AFB sputum smear negativity and complications—including fever, chest tightness, and hemoptysis—were also determined. Assuming two consecutive negative sputum culture results, the number of days taken for the first conversion of the sputum smear from positive to negative was defined as the time to SCC. The efficacy of the EBV treatment was divided into four grades, and the grading criteria were as follows: (1) ineffective, no change in cavity size; (2) effective, the size of the cavity was reduced by less than 50%; (3) markedly effective, the size of the cavity decreased by less than 100%, but more than 50%; and (4) cured, the cavity closed. The EBV was removed three months after the cavity was closed. After the EBV was removed, and SCC was performed for six months, the anti-tuberculosis drugs were stopped. Follow-up was continued for three years after the anti-tuberculosis medicines were discontinued.

### 4.6. Statistical Methods

The data were analyzed using GraphPad Prism 9.4.0 software (San Diego, CA, USA). For the enumeration data, a chi-square test or Fisher’s exact test was used according to the number of cases. The RR and OR were determined using the Koopman asymptotic score and Baptista–Pike method, respectively. Correlations between sex, age, disease course, number of EBVs, number of cavities, changes in cavity, cavity closure time, adverse events, time to SCC, linezolid use, and recurrence were assessed using Pearson’s correlation coefficient. The correlation between the cavity closure time and disease course or time to SCC was analyzed using a simple linear regression. *p* < 0.05 was considered statistically significant.

## 5. Conclusions

A one-way EBV implantation through the cavitary drainage bronchus can promote the closure of the cavity and improve the sputum negative conversion rate in patients with MDR-PTB, improving the cure rate of patients with MDR-PTB. EBV may be a safe and effective new method for the treatment of cavities in patients with MDR-PTB. This minimally invasive intervention is simple, easy to perform, and does not require surgery under a hard microscope. However, due to the small sample size of this study and the limited literature on this matter worldwide, the indications, valve removal time, and anti-tuberculosis drug treatment course should be explored and confirmed by a clinical trial with a larger sample size.

## Figures and Tables

**Table 1:** Comparisons of the cavity closure in different groups divided by the clinical parameters in 32 MDR-PTB patients.

Groups	Case	Number of Lung Cavities	Cavity Closure n (%)		OR	95% CI	RR	95% CI	*p*-Value *
			Closed	Unclosed					
Age									
≤40	26	38	27 (71.1%)	11 (28.9%)	1.6360	0.4465–6.1990	1.1840	0.7718–2.3270	0.7028
>40	6	10	6 (60.0%)	4 (40.0%)					
Gender									
Male	22	31	17 (54.8%)	14 (45.2%)	0.0759	0.0068–0.5555	0.5827	0.3969–0.8204	0.0078
Female	10	17	16 (94.1%)	1 (5.9%)					
Linezolid									
Unused	17	26	15 (57.7%)	11 (42.3%)	0.3030	0.0933–1.0490	0.7051	0.4614–1.0350	0.1179
Used	15	22	18 (81.8%)	4 (18.2%)					
Lung lobe									
Upper lobe	27	33	22 (66.7%)	11 (33.3%)	0.7273	0.2165–2.6800	0.9091	0.6274–1.4510	0.7458
Lower lobe	14	15	11 (73.3%)	4 (26.7%)					

* Data were analyzed using Fisher’s exact test. OR, odds ratio; CI, confidence interval; RR, relative risk.

**Table 2:** List of adverse events.

Type of Adverse Events	Number of Adverse Events (Times [%])	Number of EBV Removal (Times)
Fever	3 (23.1%)	0
Granulomatous	6 (46.2%)	0
Chest distress	1 (7.7%)	1
Hemoptysis	1 (7.7%)	0
EBV cannot be removed due to displacement or insertion	1 (7.7%)	0
EBV fall off	1 (7.7%)	0

## Data Availability

All of the data generated or analyzed during this study are included in this published article.

## Supplementary Materials

The following supporting information can be downloaded at: https://www.mdpi.com/article/10.3390/pathogens11080899/s1, Table S1: The detailed information for evaluating the correlations among 11 variates in patients with MDR-PTB treated with EBVs.
